# Effect of low-intensity blood flow restriction combined with high-intensity resistance training on lower limb strength and stroke effect of table tennis players

**DOI:** 10.3389/fbioe.2026.1934656

**Published:** 2026-09-30

**Authors:** Han Li, Rui Zhao, Yi Xiao

**Affiliations:** Shanghai University of Sport, Shanghai, China

**Keywords:** blood flow restriction training, high-intensity resistance training, muscle strength, stroke effect, table tennis players

## Abstract

This study aimed to investigate the effects of different strength training protocols on lower limb strength and stroke effect in table tennis players. A single-blind randomized controlled trial (RCT) design was employed. A total of 30 table tennis players were recruited and randomly assigned into three groups: the low-intensity blood flow restriction training (LI-BFRT) group, high-intensity resistance training (HI-RT) group, and combined training (CB-RT) group. All participants completed an 8-week strength training intervention with three sessions per week. Key indicators including knee peak torque, squat jump (SJ) height, countermovement jump (CMJ) height, peak rate of force development (PRFD), and stroke effect were measured before and after the intervention. No significant differences in all baseline indicators were observed among the three groups (p > 0.05). After the intervention, the CB-RT group exhibited extremely significant improvements in knee flexion and extension peak torques, SJ and CMJ heights, PRFD, and stroke effect (p < 0.01). In the LI-BFRT group, left knee extension PT was extremely significantly improved (p < 0.01); Meanwhile SJ height, PRFD, and forehand topspin loop effect were all significantly enhanced (p < 0.05). In the HI-RT group, left knee extension PT was extremely significantly increased (p < 0.01); Meanwhile, left knee flexion PT, right knee flexion and extension peak torques, CMJ height, PRFD, and forehand backspin loop effect were all significantly improved (p < 0.05). The results indicate that compared with single LI-BFRT or HI-RT, CB-RT exhibited a greater improvement trend in lower limb strength and stroke effect among table tennis players.

## Introduction

Table tennis is a net-separated and skill-dominated competitive sports characterized by short-duration explosive force generation, rapid movement and high-frequency confrontation. Frequent rapid movement, direction change, and stroke during competitions impose high requirements on the strength of athletes’ lower limb muscles (Picabea-Arburu and Ozaeta-Beaskoetxea, 2025). A table tennis stroke follows the force generation principle of kinetic chain (Gribble et al., 2009). As the initial segment of energy generation, the strength level of the lower limbs directly determines the execution quality of athletes’ key technical movements such as starting, moving, and braking, and serves as the core strength foundation for improving competitive performance (Pradas de la Fuente et al., 2023; Qian et al., 2016). Furthermore, with the continuous adjustment of the competition rules by the International Table Tennis Federation, especially the changes in the material and weight of table tennis balls, the ball speed of stroke has decreased while the number of rally in matches has increased, which imposes higher requirements for the lower limb strength quality of table tennis athletes (Picabea-Arburu and Ozaeta-Beaskoetxea, 2025).

Lower limb strength is also critical for injury prevention in table tennis players. Epidemiological evidence has demonstrated that lower limb injuries account for nearly 50% of all sports-related injuries sustained by these athletes. Frequent rapid acceleration, deceleration, and cutting movements during competitions predispose athletes to sports injuries at the knee joint and other lower limb joints (Lambert et al., 2025). Meanwhile, chronic knee strain and osteoarthritis are easily induced by long-term inappropriate loading stimuli and insufficient muscular protective strength, which severely undermine athletes’ training continuity and athletic career longevity (Biz et al., 2022). Accordingly, scientific lower limb resistance training can not only improve stroke effect but also deliver effective injury prevention, conferring dual practical benefits for enhancing athletes’ competitive capacity and training safety.

In practical strength training, traditional high-intensity resistance training (HI-RT, 60%–80% of one repetition maximum [1RM]) serves as the primary method for developing lower limb muscle strength. This training method involves muscle contraction exercises performed by actively overcoming external resistance, such as equipment load, resistance band tension, and body weight. It can effectively enhance muscular strength, muscle hypertrophy, and neuromuscular adaptation (Sarfabadi et al., 2023; Zhang et al., 2024). The American College of Sports Medicine (ACSM) has pointed out that traditional strength training can effectively promote the development of muscle strength only when the training load intensity reaches or exceeds 70% 1RM, where 1RM is defined as the maximum load an individual can complete with one single standard repetition (American College of Sports Medicine, 2009). Nevertheless, long-term engagement in HI-RT renders athletes vulnerable to sports injuries (Yang et al., 2024). Meanwhile, table tennis athletes need to develop multiple physical fitness qualities simultaneously. Repeated HI-RT not only induces cumulative muscle fatigue, but may also interfere with the effectiveness of specialized technical training. These limitations have prompted researchers and coaches to gradually explore safer and more efficient alternative training modes. In recent years, numerous studies have confirmed that low-intensity blood flow restriction training (LI-BFRT, 20%–40% 1RM, 15–30 repetitions per set) can achieve effects comparable to traditional HI-RT in improving athletes’ muscle strength and muscle mass. Additionally, due to its low training load intensity, this training method can significantly reduce the risk of sports injuries (Lixandrão et al., 2018).

Blood flow restriction training (BFRT) is a specialized training modality that involves placing pressure cuffs on the proximal segments of the limbs to exert external compressive pressure. This intervention partially restricts venous blood return while maintaining normal arterial blood inflow, thereby increasing the accumulation of intramuscular metabolic and acidic metabolites and exacerbating local muscle fatigue, which ultimately contributes to the improvement of muscle strength (Jessee et al., 2019).

LI-BFRT has been widely applied in competitive sports, as this training method can effectively improve the muscular strength of elite athletes. Luebbers conducted a 7-week intervention experiment with 72 rugby players as research subjects. The results revealed that the participants in the BFRT group achieved significantly greater strength compared with those in the conventional non-BFRT group (Luebbers et al., 2014). Miller et al. further demonstrated that LI-BFRT not only enhances participants’ maximal strength, but also significantly improves their countermovement jump (CMJ) performance, indicating that this training method also exerts a positive effect on the improvement of athletes’ explosive force (Miller et al., 2018).

Traditional HI-RT primarily induces muscular adaptive responses by imposing high mechanical tension on skeletal muscles (Crewther et al., 2005), with its core training effects focused on the improvement of maximal muscle strength and neuromuscular adaptation. In contrast, LI-BFRT induces metabolic stress by restricting limb blood flow under low-load conditions, thereby promoting the secretion of growth hormones and further inducing muscle hypertrophy. This training approach can achieve training effects comparable to those of HI-RT and is more conducive to the development of athletes’ explosive force (Pearson and Hussain, 2015). Nevertheless, each single training approach exhibits inherent limitations in practical application. Long-term isolated HI-RT imposes excessive mechanical stress on muscles and joints, which tends to induce cumulative muscle fatigue and sports injuries (Yang et al., 2024). Furthermore, its high-load intervention may compromise the technical training effectiveness of table tennis-specific skills. In contrast, isolated LI-BFRT fails to provide sufficient mechanical tension stimuli, resulting in limited improvements in athletes’ lower limb maximal strength. Recent investigations have revealed that combined training (CB-RT), which integrates LI-BFRT and HI-RT, enables complementary advantages of the two training protocols and thereby yields superior overall training outcomes. Accumulating evidence has verified that CB-RT elicits more favorable muscular adaptive responses than isolated LI-BFRT or HI-RT alone, and substantially enhances athletes’ lower limb strength and athletic performance. For instance, Gan’s study demonstrated that eight weeks of combined HI-RT and LI-BFRT significantly improved the sprint acceleration and approach run capacities of basketball players (Gan and Hui, 2019). Similarly, Jiang’s research indicated that this CB-RT protocol produced significantly better effects on the improvement of lower limb muscle strength, vertical jump performance, and the rate of force development (RFD) in adolescent female wrestlers compared with isolated HI-RT or single LI-BFRT (Jiang et al., 2025). In addition, multimodal resistance training combining high and low intensities can elicit stronger muscular adaptive changes than single-intensity resistance training (Goto et al., 2004).

In summary, CB-RT has been verified to effectively improve athletes’ lower limb muscle strength and athletic performance while reducing sports-specific injury risks. Nevertheless, relevant research focusing on table tennis remains scarce. Furthermore, existing studies fail to integrate the distinct adaptive characteristics of different training protocols with the force-generation patterns of table tennis techniques, making it difficult to elucidate the intrinsic correlation between enhanced lower limb strength to improved stroke effect. Accordingly, this study aimed to investigate the effects of an 8-week intervention with three different strength training protocols on lower limb strength and stroke effect of forehand loop in table tennis players. The findings provide experimental evidence and practical training references for the scientific, efficient, and low-injury development of lower limb strength and the improvement of stroke effect of table tennis players. The hypotheses of the present study were proposed as follows:

1. Following an 8-week training intervention, significant improvements in knee isokinetic muscle strength, jump performance, and stroke effect were observed in the CB-RT group, LI-BFRT group, and HI-RT group;
2. Compared with the LI-BFRT group and HI-RT group, the CB-RT group demonstrated greater magnitudes of improvement in all the above indicators.

## Methods

### Participants

A total of 24 participants were needed for this study to reach a power of 80% at an alpha level of 0.05 based on a statistical power analysis to detect an effect size of ES = 0.25 (Faul et al., 2009). A total of thirty table tennis players (15 males and 15 females) were recruited from the China Table Tennis College (age (yr): 21.47 ± 2.20, stature (cm): 173.77 ± 8.91, mass (kg): 65.97 ± 10.14, BMI (kg/m2): 21.75 ± 2.07, training year (yr): 13.47 ± 2.6). The participants were randomly assigned into three groups, including the low-intensity blood flow restriction training (LI-BFRT) group (n = 10), the high-intensity resistance training (HI-RT) group (n = 10), and the combined training (CB-RT) group (n = 10). After randomization, each group comprised 5 male and 5 female participants, ensuring a balanced sex distribution across groups. Subject recruitment commenced on 23 February 2026, and was completed on 27 February 2026 (see Figure 1 for the recruitment procedure). The inclusion criteria were as follows: table tennis athletes with technical levels of National Grade one or above, right-handed (dominant hand), aged 18–28 years, good physical health without underlying chronic diseases, no history of sports injuries in the past one year, and capable of completing moderate-intensity and above training. The exclusion criteria included: participants with cardiovascular diseases, neurological injuries, or somatosensory dysfunctions (Sarfabadi et al., 2023); individuals with a history of lower limb surgery and any other physical conditions or sports injuries that might affect high-intensity exercise training. Basic demographic information of all participants was presented in Table 1. This study was conducted in accordance with the principles of the Declaration of Helsinki and approved by the Ethics Review Committee of Shanghai University of Sport (Approval No. 102772025RT177). Prior to the experiment, all participants were fully informed of the research content, experimental procedures, potential risks, and research benefits, and written informed consent was obtained from each participant.

**FIGURE 1:**
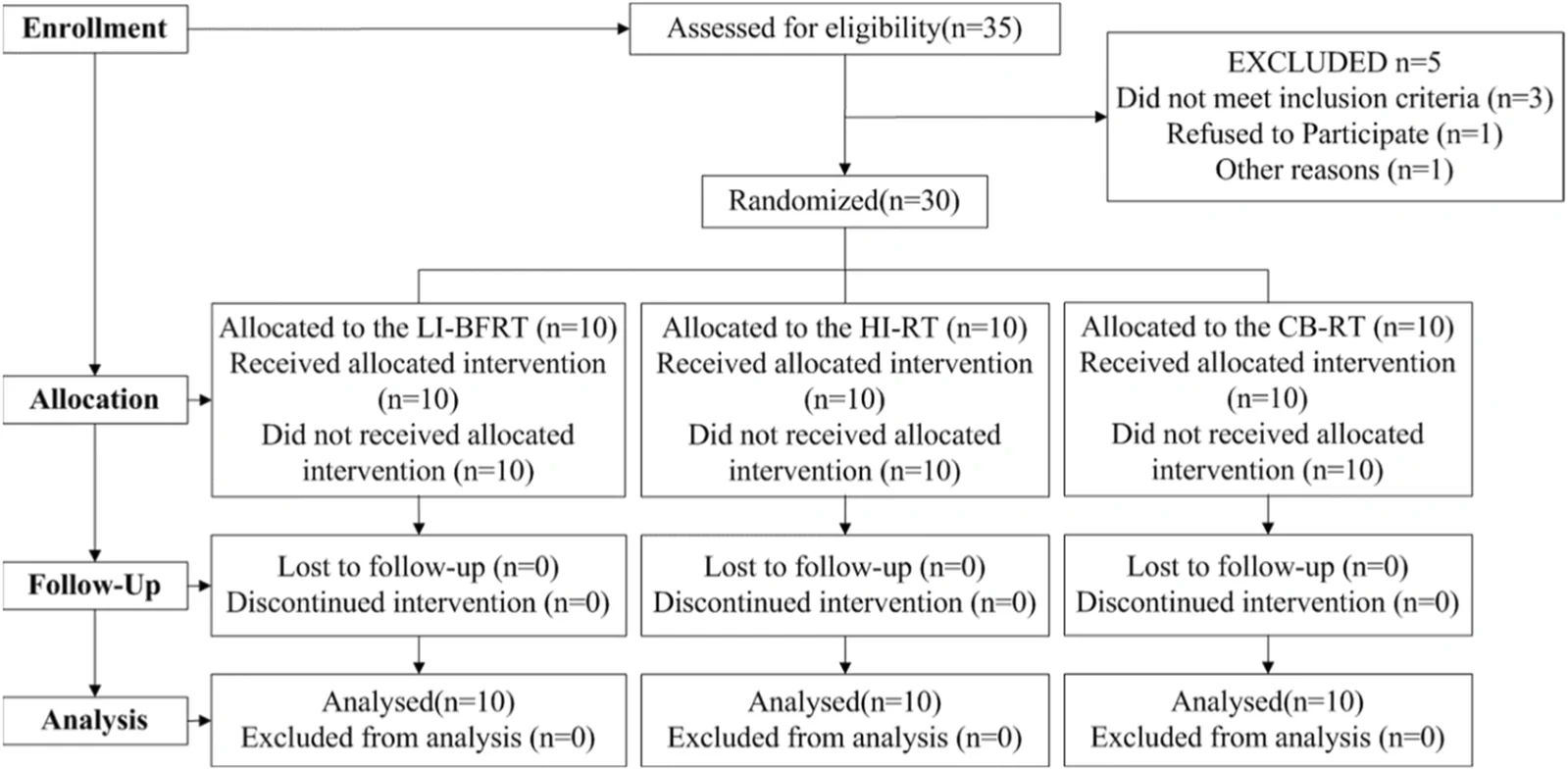
Flow chart of the progress through the phases of the study according to the consort statements. Note: Adherence rate reached 100% across all participants (all 24 training sessions completed); zero participants were lost to follow-up, and no protocol deviations or intervention-related injuries occurred during the 8-week trial.

**TABLE 1:** Basic information of subjects.

Group	Age	Height (cm)	Weight (kg)	BMI (kg/m^2^)	Training years (years)
LI-BFRT	22 ± 2.54	173.9 ± 9.26	67.1 ± 10.55	22.14 ± 2.62	14.3 ± 2.26
HI-RT	20.3 ± 1.42	176.9 ± 8.82	67.9 ± 10.89	21.57 ± 1.87	11.7 ± 2.49
CB-RT	22.1 ± 2.65	170.5 ± 8.66	62.9 ± 8.99	21.55 ± 1.71	14.4 ± 3.06

BMI, body mass index.

### Study design and blinding

This study adopted a randomized controlled trial (RCT) design to explore the comprehensive effects of CB-RT. This study was conducted as a single-blind trial, in which only the outcome assessors were blinded to group allocation to minimize experimental bias. Participants knew which intervention they were receiving. Participants were randomized at a 1:1:1 ratio using computer-generated numbers. The allocation sequence was concealed in sequentially numbered, opaque, and sealed envelopes. One researcher was responsible for participant recruitment and baseline testing, whereas another researcher uninvolved in enrolment opened the envelopes and allocated participants to three distinct groups upon completion of baseline assessments. This separation of roles minimized selection bias and ensured the randomness and objectivity of group allocation. All tests and training interventions in this study were conducted in the training hall on the third floor of the China Table Tennis College, ensuring the consistency and standardization of the experimental environment.

## Experimental protocol

This study was conducted in three phases: the pre-test was completed from March 2 to 6 March 2026; an 8-week training intervention was implemented from March 3 to 1 May 2026; the post-test was finished from May 4 to 8 May 2026.

The first stage was the pre-test, which collected basic information of all subjects such as height, weight, and years of training, and conducted tests including 1 R M squat test, lower limb maximal strength, lower limb explosive force and stroke effect, so as to provide baseline data for the implementation of the subsequent intervention program and the comparison of post-test results.

The second stage was the intervention period. All three groups of participants underwent daily table tennis training, which included forehand and backhand drives, forehand and backhand loops, random full-court loops, attack after serve, and attack after receive. All groups followed identical daily training schedules, ensuring consistent training volume and intensity. In addition to routine table tennis-specific training, all three groups of participants underwent strength training intervention for 8 weeks, 3 times a week, with 30 min per session (Rong et al., 2025; Yang et al., 2022). Throughout this period, participants were instructed not to engage in any additional physical or strength training beyond the prescribed intervention protocol. Training attendance was recorded for each session, and all participants demonstrated 100% adherence with no drop-outs. The intervention content for all three groups adopted squat, deadlift and lunge exercises. Based on previous research findings on the dose-response relationship of BFRT, the training load intensity of the LI-BFRT group in this study was set at 30% 1 R M. Each of the three exercise movements was repeated 75 times, divided into 4 sets (30 repetitions in the first set and 15 repetitions each in the 2nd to 4th sets) with a 60-s rest interval between sets, and the lower limbs maintained a pressurized state throughout the training (Yang et al., 2022). Referring to previous research results on the dose-response relationship of HI-RT, the training load intensity of the HI-RT group was set at 70% 1 R M. Each of the three exercise movements was repeated 34 times without blood flow restriction, specifically divided into 4 sets (10 repetitions in the first set and 8 repetitions each in the 2nd to 4th sets) with a 120-s rest interval between sets (Rong et al., 2025). The CB-RT group adopted a training protocol combining LI-BFRT and HI-RT, with athletes completing 2 sessions of LI-BFRT and 1 session of HI-RT per week. The detailed intervention protocols for the three groups were shown in Table 2. During the pressurized training, a Japanese-made KAATSU Master pressurizer and pressurized belts were used to exert pressure at the proximal one-third of the bilateral thighs. The cuff width was 5 cm, with an initial binding pressure of 40 mm of mercury. During the intervention period, the cuff pressure is set at 50% of the estimated arterial occlusion pressure (AOP) for each participant, which is determined based on their thigh circumference and ranges from 100 to 180 mmHg (Libardi et al., 2024).

**TABLE 2:** Design scheme of intervention training.

Groups	BFR	Load	Times/week	Sets/time	Reps/set	Interval between sets (s)
LI-BFRT	√	30% 1 R M	3	4	Set 1: 30 reps Sets 2 to 4: 15 reps/set	60s
HI-RT	×	70% 1 R M	3	4	Set 1: 10 reps Sets 2 to 4: 8 reps/set	120s
CB-RT	√	30% 1 R M	2	4	Set 1: 30 reps Sets 2 to 4: 15 reps/set	60s
	×	70% 1 R M	1	4	Set 1: 10 reps Sets 2 to 4: 8 reps/set	120s

BFR, Blood Flow Restriction. “√” indicates that BFR, was used during the training, while “×” indicates that BFR, was not used.

The third phase was the post-test. All three groups repeated the content of the pre-test using the same instruments, methods, and s criteria to ensure the authenticity and comparability of the pre- and post-test data.

### Experimental test indicators

The lower limb strength and stroke effect of the three groups of athletes were tested before and after the intervention. Specifically, maximal lower limb strength was assessed using the knee isokinetic muscle strength test (Wang et al., 2020), lower limb explosive force was comprehensively evaluated through the SJ and CMJ tests (Radakovic et al., 2025), and stroke effect was measured using ball speed and placement (Li et al., 2025). The experimental testing procedure was illustrated in Figure 2.

**FIGURE 2:**
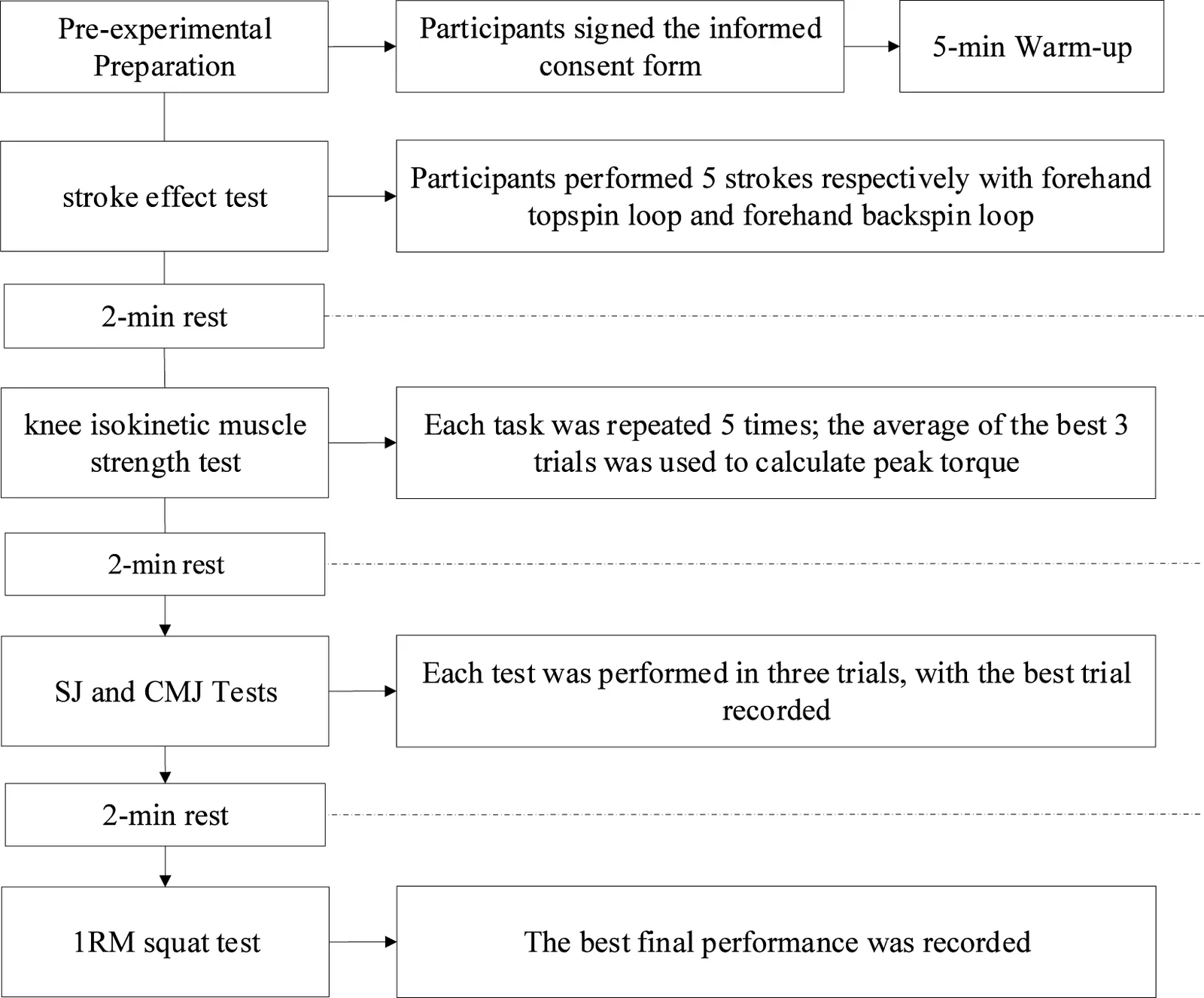
Experimental test flow chart.

### Stroke effect test

During the test of stroke effect, each participant was required to perform five consecutive high-quality strokes using two typical techniques: the forehand topspin loop and forehand backspin loop. A table tennis serving machine (Pongbot, China) delivered topspin and backspin balls at frequencies of 40 balls per minute and 25 balls per minute, respectively (Tian and Xiao, 2024). The SEEKER smart table tennis hawk-eye system (Pongbot, China) was adopted to measure the ball speed of each stroke. To evaluate the stroke accuracy, five rectangular target areas with sizes ranging from 10 cm × 10 cm–50 cm × 50 cm were marked on the left baseline area of the table tennis table, corresponding to scoring values from 1 to 5 points. A smaller target area represents a higher score, and a higher score indicates better stroke accuracy. The experimental test setup was shown in Figure 3.

**FIGURE 3:**
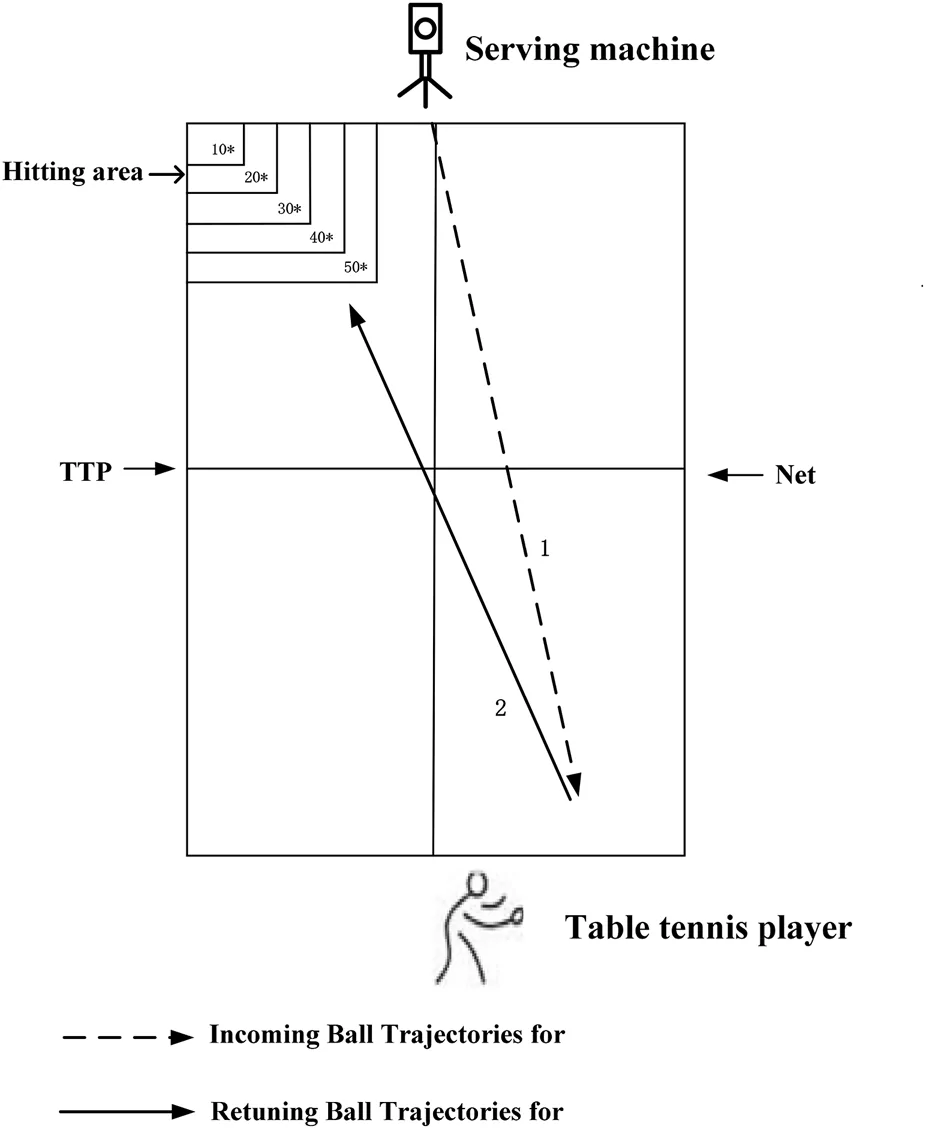
Experimental set-up of the ball speed and accuracy.

### Knee joint isokinetic muscle strength measurement

In this study, an isokinetic strength testing system (ISOMED 2000, Germany) was used to test the peak torque of knee flexion and extension movements in all subjects (Wang et al., 2020).

Before the formal test, subjects completed 20 low-speed concentric-eccentric alternating exercises at an angular velocity of 15/s for warm-up. The formal test adopted the concentric-eccentric contraction mode, with a knee joint range of motion of 70° (extending from 80° flexion to 10° flexion) and an angular velocity of 60/s. Each movement was repeated 5 times, and the average value of the three best trials was used to calculate the peak torque. The peak torque refers to the maximum torque generated during muscle contraction, which reflects the maximal strength produced by muscle contraction (Wang et al., 2020), with the unit of Nm.

### Vertical jump test

In this study, the squat jump (SJ) and countermovement jump (CMJ) tests were performed to evaluate the intervention effect of strength training on the concentric contraction explosive force and the explosive force during the rapid eccentric-to-concentric contraction transition of lower limb muscles in table tennis players. Jump height and the rate of force development (RFD) were selected as the core evaluation indicators (Mc et al., 2011).

During the SJ test, participants stood on a one-dimensional Kistler force platform with a neutral spinal posture and hands placed on the hips. After maintaining a static squat position with the knee joint flexed at 90° for 3 seconds, participants were required to jump vertically upward as rapidly as possible without any countermovement. For the CMJ test, participants also stood on the force platform with a neutral spine posture and hands on the hips. Following the test instruction, participants rapidly performed a downward squat until the knee joint reached approximately 90° flexion, and then immediately jumped vertically in an explosive manner, utilizing the countermovement to achieve the maximum jump height. Each test was repeated three times, with a 1-min rest interval between trials. The best performance among the three trials was selected as the final test data.

### 1RM squat measurement

For the measurement of 1 R M squat, participants were asked for the predicted weight by which they can complete only one squat for full range-of-motion (ROM) (Meechan et al., 2020). Participants stood on a Smith machine with their feet shoulder-width apart. The squatted down until the knee joint angle reached 60°–70° (thighs parallel to the ground), and returned to an upright position by synchronous extension of the hip and knee joints. A progressive load-increment protocol was adopted throughout the 1 R M test. Participants first completed five squat repetitions at 50% of the estimated 1 R M, followed by three repetitions at 60% and two repetitions at 80% of the estimated 1 R M. Subsequently, single attempts were performed sequentially at 90%, 95%, and 100% of the estimated 1 R M. Upon successful completion of the 100% estimated 1 R M, the external load was gradually increased by 2.5 kg per attempt until failure (Sarfabadi et al., 2023). On average, each participant completed three maximal attempts to determine the final actual 1 R M value, with a 5-min rest interval provided between each attempt. The standardized movement requirements and technical specifications for the 1 R M squat test were fully explained and demonstrated to all participants prior to the formal testing.

### Statistical analysis

Data analysis was performed using SPSS 26. Data were normally distributed. Descriptive statistics were used to calculate the mean and standard deviation for all variables. A 3 × 2 (group × time) mixed repeated-measures analysis of variance (ANOVA) was used to examine the main effects of group and time, and the interaction effect for all observed variables, so as to analyze the differential characteristics of each indicator among different groups and at different time points. Bonferroni correction was applied to the between-group post-hoc comparisons within each outcome measure, and adjusted p-values were reported for post-hoc pairwise comparisons. A probability level of less than 0.05 (p < 0.05) was considered significant in all statistical analyses. Cohen’s d was adopted to quantify the magnitude of intervention effects. Effect sizes were categorized as small effect size (0.2 ≤ d < 0.5), medium effect size (0.5 ≤ d < 0.8), and large effect size (d ≥ 0.8). The 95% confidence intervals for mean changes were also calculated and reported. To intuitively reflect the changes in indicators before and after intervention, the “Change ratio' indicator was introduced. It was calculated as the difference between post- and pre-intervention values divided by the pre-intervention value. A change ratio greater than 0 indicates an improvement in the indicator, and the larger the value, the greater the range of improvement.

## Results

A 3 (Group: CB-RT, LI-BFRT, HI-RT) × 2 (Time: pre-test, post-test) repeated measures analysis of variance (ANOVA) was conducted for statistical analysis of test indicators including knee isokinetic muscle strength, jump performance, and stroke performance.

### Comparison of knee joint isokinetic muscle strength

The results of knee joint peak torque of participants in the three groups before and after intervention were presented in Table 3. Prior to the intervention, there were no significant differences in the four indicators among the three groups (p > 0.05), indicating that all groups had consistent baseline levels and good comparability. Significant group × time interaction effects were observed for all four peak torque variables (F (Aram et al., 2024; Miller et al., 2018) = 3.604–4.606, p = 0.019–0.041, partial η^2^ = 0.211–0.254). After an 8-week strength training intervention implemented three times per week, the HI-RT group showed significant pre-post differences in peak torque of the right knee flexion (Cohen’s d = 0.36), right knee extension (Cohen’s d = 0.16), and left knee flexion (Cohen’s d = 0.45) (p < 0.05), while the peak torque of left knee extension (Cohen’s d = 0.33) exhibited an extremely significant difference (p < 0.01). In the LI-BFRT group, only the peak torque of left knee extension (Cohen’s d = 0.38) had a significant pre-post improvement (p < 0.01); the peak torque of right knee flexion (Cohen’s d = 0.20), right knee extension (Cohen’s d = 0.18), and left knee flexion (Cohen’s d = 0.16) increased slightly but did not reach the statistically significant level (p > 0.05). The CB-RT group showed extremely significant pre-post differences in all four indicators (p < 0.01). Cohen’s d for peak torque was 0.46 (right knee flexion), 0.61 (right knee extension), 1.04 (left knee flexion), and 0.45 (left knee extension), with the corresponding indicator change ratio being 13.79%, 17.20%, 17.01% and 14.05%, respectively. After the intervention, post-hoc pairwise comparisons revealed no significant inter-group differences in any outcome measure among the three groups (adjusted p > 0.05), except for left knee flexor peak torque (adjusted p = 0.030), which was significantly different between the CB-RT and LI-BFRT groups (Table 4).

**TABLE 3:** Pre- and post-test results of the peak torques of knee joint flexion and extension in three groups.

Variable	Group	Pre-test	Post-test	*p*-value	Interaction effect			Cohen’s d	95% CI		Change ratio (%)
					F (Aram et al., 2024; Miller et al., 2018)	p-value	partial η^2^		Lower	Upper	
Flexors(R) (nm)	CB-RT	59.76 ± 13.09	68.00 ± 21.51	0.000**	3.604	0.041*	0.211	0.46	−12.13	−4.337	13.79%
	LI-BFRT	61.06 ± 19.05	64.86 ± 18.67	0.056				0.20	−7.697	0.097	6.22%
	HI-RT	61.96 ± 11.15	65.96 ± 11.35	0.044*				0.36	−7.897	−0.103	6.46%
Extensors(R) (nm)	CB-RT	128.93 ± 33.40	151.10 ± 38.81	0.000**	3.734	0.037*	0.217	0.61	−28.615	−15.719	17.20%
	LI-BFRT	132.43 ± 32.14	134.96 ± 34.80	0.437				0.08	−8.981	3.915	1.91%
	HI-RT	129.20 ± 44.86	135.86 ± 40.78	0.043*				0.16	−13.115	−0.219	5.15%
Flexors(L) (nm)	CB-RT	58.37 ± 8.92	68.30 ± 10.11	0.000**	4.048	0.029*	0.231	1.04	−15.135	−4.732	17.01%
	LI-BFRT	56.73 ± 16.15	59.26 ± 16.22	0.336				0.16	−7.735	2.668	4.46%
	HI-RT	55.40 ± 13.17	61.27 ± 12.88	0.028*				0.45	−11.068	−0.665	10.59%
Extensors(L) (nm)	CB-RT	133.13 ± 42.32	151.83 ± 40.73	0.000**	4.606	0.019*	0.254	0.45	−26.717	−10.683	14.05%
	LI-BFRT	120.33 ± 32.46	132.76 ± 32.81	0.003**				0.38	−20.45	−4.417	10.33%
	HI-RT	117.93 ± 41.22	132.00 ± 44.99	0.001**				0.33	−32.083	−16.05	11.93%

(R) represents the right knee, (L) represents the left knee. Pre-, before training; Post-, after training; *p < 0.05, **p < 0.01.

**TABLE 4:** Between-group post-hoc pairwise comparisons for knee flexion and extension peak torque among the three groups.

Variable	Comparison	Δ Mean difference	95% CI		Adjusted p-value	Cohen’s d
			Lower	Upper		
Flexors(R) (nm)	CB-RT vs. LI-BFRT	4.44	−8.026	14.293	0.884	0.16
	CB-RT vs. HI-RT	4.24	−9.126	13.193	0.907	0.12
	LI-BFRT vs. HI-RT	−0.20	−12.26	10.06	0.986	−0.07
Extensors(R) (nm)	CB-RT vs. LI-BFRT	17.32	−7.955	40.221	0.317	0.44
	CB-RT vs. HI-RT	6.47	−8.855	39.321	0.379	0.38
	LI-BFRT vs. HI-RT	−10.85	−24.988	23.188	0.873	−0.02
Flexors(L) (nm)	CB-RT vs. LI-BFRT	8.51	0.654	17.422	0.030*	0.67
	CB-RT vs. HI-RT	5.26	−1.355	15.422	0.131	0.61
	LI-BFRT vs. HI-RT	−3.25	−10.389	6.389	0.942	−0.14
Extensors(L) (nm)	CB-RT vs. LI-BFRT	4.98	−6.043	44.176	0.202	0.52
	CB-RT vs. HI-RT	4.02	−15.276	34.943	0.911	0.46
	LI-BFRT vs. HI-RT	−0.96	−34.343	15.876	0.945	0.02

(R) represents the right knee, (L) represents the left knee. *p < 0.05, **p < 0.01.

### Comparison of vertical jump height

The pre- and post-test results of SJ and CMJ vertical jump heights for participants in the three groups were shown in Table 5. Before the intervention, there were no significant differences in SJ and CMJ vertical jump heights among the three groups (p > 0.05). Significant group × time interaction effects were observed for both SJ height (F (Aram et al., 2024; Miller et al., 2018) = 3.432, p = 0.047, partial η^2^ = 0.203) and CMJ height (F (Aram et al., 2024; Miller et al., 2018) = 3.543, p = 0.043, partial η^2^ = 0.208). After an 8-week strength training intervention conducted three times per week, the LI-BFRT group showed a significant pre-post difference only in SJ vertical jump height (Cohen’s d = 0.15, p < 0.05), with a change ratio of 4.12%. The HI-RT group exhibited a significant pre-post difference only in CMJ vertical jump height (Cohen’s d = 0.34, p < 0.05), with a change ratio of 5.31%. In the CB-RT group, both SJ (Cohen’s d = 0.24) and CMJ (Cohen’s d = 0.37) vertical jump heights presented extremely significant pre-post differences (p < 0.01), with change ratio of 5.71% and 7.77% respectively, representing the greatest magnitude of improvement among three groups. Post-hoc pairwise comparisons revealed no significant differences in SJ and CMJ vertical jump heights were found among the three groups after the intervention (adjusted p > 0.05) (Table 6). Overall, the CB-RT training protocol demonstrated a greater magnitude of improvement effects.

**TABLE 5:** Pre- and post-test results of SJ and CMJ vertical jump heights in three groups.

Variable	Group	Pre-test	Post-test	*p*-value	Interaction effect			Cohen’s d	95% CI		Change ratio (%)
					F (Aram et al., 2024; Miller et al., 2018)	p-value	partial η^2^	​	Lower	Upper	​
SJ-height (cm)	CB-RT	26.09 ± 6.83	27.58 ± 5.60	0.004**	3.432	0.047*	0.203	0.24	−2.49	−0.476	5.71%
	LI-BFRT	26.21 ± 7.60	27.29 ± 6.85	0.035*				0.15	−2.09	−0.076	4.12%
	HI-RT	26.20 ± 4.67	26.73 ± 4.31	0.305				0.12	−1.53	0.484	2.02%
CMJ-height (cm)	CB-RT	28.04 ± 6.55	30.22 ± 5.20	0.003**	3.543	0.043*	0.208	0.37	−3.547	−0.786	7.77%
	LI-BFRT	27.64 ± 5.73	28.07 ± 4.26	0.540				0.09	−1.82	0.960	1.56%
	HI-RT	27.85 ± 4.75	29.33 ± 3.96	0.038*				0.34	−2.867	−0.086	5.31%

Pre-, before training; Post-, after training; *p < 0.05, **p < 0.01.

**TABLE 6:** Between-group post-hoc pairwise comparisons for SJ and CMJ vertical jump heights among the three groups.

Variable	Comparison	Δ Mean difference	95% CI		Adjusted p-value	Cohen’s d
			Lower	Upper		
SJ-height (cm)	CB-RT vs. LI-BFRT	0.41	−3.300	3.866	0.941	0.05
	CB-RT vs. HI-RT	0.96	−2.733	4.433	0.893	0.17
	LI-BFRT vs. HI-RT	0.55	−3.016	4.150	0.927	0.10
CMJ-height (cm)	CB-RT vs. LI-BFRT	1.75	−0.697	4.990	0.206	0.45
	CB-RT vs. HI-RT	0.70	−1.953	3.733	0.924	0.19
	LI-BFRT vs. HI-RT	−1.05	−4.100	1.587	0.851	−0.31

*p < 0.05, **p < 0.01.

### Comparison of PRFD during take-off of vertical jump

The pre- and post-intervention test results of PRFD during the take-off phase in the three groups were presented in Table 7. Significant group × time interaction effects were observed for SJ-PRFD (F (Aram et al., 2024; Miller et al., 2018) = 4.240, p = 0.025, partial η^2^ = 0.239) and CMJ-PRFD (F (Aram et al., 2024; Miller et al., 2018) = 3.921, p = 0.032, partial η^2^ = 0.225). In the LI-BFRT group, only the PRFD during the SJ take-off phase was significantly improved (Cohen’s d = 0.31, p < 0.05), with an increase of 7.01%. In the HI-RT group, significant improvement was observed only in the PRFD during the CMJ take-off (Cohen’s d = 0.24, p < 0.05), with an increment of 6.96%. The CB-RT group showed the highest growth magnitude, with PRFD change ratio of 7.94% and 10.13% for the SJ and CMJ take-off phases, respectively. In the CB-RT group, PRFD during the SJ (Cohen’s d = 0.36, p < 0.05) and CMJ (Cohen’s d = 0.32, p < 0.01) take-off were significantly enhanced. Post-hoc pairwise comparisons revealed no significant inter-group differences in PRFD during the SJ and CMJ take-off were detected among the three groups after the intervention (adjusted p > 0.05) (Table 8).

**TABLE 7:** Pre- and post-test results of PRFD during take-off in the three groups.

Variable	Group	Pre-test	Post-test	*p*-value	Interaction effect			Cohen’s d	95% CI		Change ratio (%)
					F (Aram et al., 2024; Miller et al., 2018)	p-value	partial η^2^		Lower	Upper	
SJ-PRFD (N/S)	CB-RT	12767.50 ± 2,687.80	13781.73 ± 2,919.84	0.017*	4.240	0.025*	0.239	0.36	−1844.747	−183.72	7.94%
	LI-BFRT	12432.73 ± 2,549.57	13304.56 ± 3,019.99	0.04*				0.31	−1702.347	−41.32	7.01%
	HI-RT	12139.26 ± 2,570.72	12442.70 ± 2,353.79	0.47				0.12	−1,133.947	527.08	2.49%
CMJ-PRFD (N/S)	CB-RT	14526.93 ± 4,976.72	15998.23 ± 4,254.19	0.001**	3.921	0.032*	0.225	0.32	−2,312.059	−630.541	10.13%
	LI-BFRT	14490.86 ± 5,029.67	14767.26 ± 3,681.65	0.515				0.06	−1,117.159	564.359	1.91%
	HI-RT	14375.86 ± 3,900.95	15376.03 ± 4,472.23	0.02*				0.24	−1840.925	−159.408	6.96%

PRFD, peak rate of force development. Pre-, before training; Post-, after training; *p < 0.05, **p < 0.01.

**TABLE 8:** Between-group post-hoc pairwise comparisons for PRFD during take-off among the three groups.

Variable	Comparison	Δ Mean difference	95% CI		Adjusted p-value	Cohen’s d
			Lower	Upper		
SJ-PRFD (N/S)	CB-RT vs. LI-BFRT	142.4	−1,275.112	2,229.446	0.915	0.16
	CB-RT vs. HI-RT	710.79	−413.246	3,091.412	0.196	0.50
	LI-BFRT vs. HI-RT	568.39	890.412	2,614.146	0.699	0.32
CMJ-PRFD (N/S)	CB-RT vs. LI-BFRT	1,194.9	−1,384.438	3,846.371	0.761	0.31
	CB-RT vs. HI-RT	471.13	−1,339.205	3,237.605	0.921	0.14
	LI-BFRT vs. HI-RT	−723.77	−3,224.171	2006.638	0.918	−0.15

PRFD, peak rate of force development. *p < 0.05, **p < 0.01.

### Pre- and post-test results of forehand topspin loop effect in three groups

The pre- and post-test results of stroke effect of forehand topspin loop in the three groups were shown in Table 9. Before the intervention, there were no significant inter-group differences in placement score and ball speed among the three groups (p > 0.05). Significant group × time interaction effects were observed for placement score (F (2, 147) = 3.167, p = 0.045, partial η^2^ = 0.041) and ball speed (F (2, 147) = 3.492, p = 0.033, partial η^2^ = 0.045). After an 8-week strength training intervention performed three times per week, the LI-BFRT group exhibited significant pre-post improvements in placement score (Cohen’s d = 0.59) and ball speed (Cohen’s d = 0.42) (p < 0.01), with change ratio of 32.8% and 6.34%, respectively. Although the HI-RT group showed no statistically significant pre-post differences in placement score (Cohen’s d = 0.29) and ball speed (Cohen’s d = 0.14) (p > 0.05), both indicators presented an upward trend with change ratio of 16.94% and 2.01%, respectively, indicating a certain training effect. The CB-RT group demonstrated significant pre-post differences in both placement score (Cohen’s d = 0.64) and ball speed (Cohen’s d = 0.68) (p < 0.01), with change ratio of 37.19% and 8.61%, respectively, showing the largest improvement magnitude among three groups. Post-hoc pairwise comparisons revealed no significant inter-group differences in placement score and ball speed were observed among the three groups after the intervention (adjusted p > 0.05) (Table 10).

**TABLE 9:** Pre- and post-test results of forehand topspin loop effect in the three groups.

Variable	Group	Pre-test	Post-test	*p*-value	Interaction effect			Cohen’s d	95% CI		Change ratio (%)
					F (Aram et al., 2024; 147)	p-value	partial η^2^		Lower	Upper	
Placement (points)	CB-RT	2.42 ± 1.35	3.32 ± 1.46	0.002**	3.167	0.045*	0.041*	0.64	−1.451	−0.349	37.19%
	LI-BFRT	2.50 ± 1.43	3.32 ± 1.30	0.004**				0.59	−1.371	−0.269	32.8%
	HI-RT	2.36 ± 1.41	2.76 ± 1.37	0.154				0.29	−0.951	0.151	16.94%
Ball speed (m/s)	CB-RT	13.11 ± 1.71	14.24 ± 1.60	0.000**	3.492	0.033*	0.045	0.68	−1.619	−0.642	8.61%
	LI-BFRT	13.23 ± 2.24	14.07 ± 1.76	0.001**				0.42	−1.331	−0.354	6.34%
	HI-RT	13.46 ± 2.24	13.73 ± 1.70	0.284				0.14	−0.754	0.223	2.01%

Pre-, before training; Post-, after training; *p < 0.05, **p < 0.01.

**TABLE 10:** Between-group post-hoc pairwise comparisons for forehand topspin loop effect among the three groups.

Variable	Comparison	Δ Mean difference	95% CI		Adjusted p-value	Cohen’s d
			Lower	Upper		
Placement (points)	CB-RT vs. LI-BFRT	0.08	−0.669	0.669	0.998	0.00
	CB-RT vs. HI-RT	0.50	−0.109	1.229	0.134	0.40
	LI-BFRT vs. HI-RT	0.42	−0.109	1.229	0.134	0.42
Ball speed (m/s)	CB-RT vs. LI-BFRT	0.29	−0.647	0.991	0.981	0.10
	CB-RT vs. HI-RT	0.86	−0.303	1.335	0.387	0.31
	LI-BFRT vs. HI-RT	0.57	−0.475	1.163	0.931	0.20

*p < 0.05, **p < 0.01.

### Pre- and post-test results of forehand backspin loop effect in three groups

The pre- and post-test results of stroke effect of forehand backspin loop in the three groups were shown in Table 11. Before the intervention, there were no significant differences in placement score and ball speed among the three groups (p > 0.05). Significant group × time interaction effects were observed for placement score (F (2, 147) = 5.579, p = 0.005, partial η^2^ = 0.071) and ball speed (F (2, 147) = 16.844, p < 0.001, partial η^2^ = 0.186). After an 8-week strength training intervention conducted three times per week, the HI-RT group achieved significant improvements in placement score (Cohen’s d = 0.53) and ball speed (Cohen’s d = 0.49) (p < 0.01), with change ratio of 22.01% and 5.93%, respectively. Although the LI-BFRT group did not reach statistical significance for placement score (Cohen’s d = 0.43) and ball speed (Cohen’s d = 0.20) (p > 0.05), both indicators showed an upward trend with change ratio of 12.72% and 2.21%, respectively, still reflecting a certain training benefit. The CB-RT group exhibited extremely significant pre-post differences in both placement score (Cohen’s d = 1.19) and ball speed (Cohen’s d = 1.47) (p < 0.01), with change ratio of 45.61% and 18.06%, respectively, showing the most prominent improvement magnitude among three groups. After the intervention, post-hoc pairwise comparisons revealed that the CB-RT group achieved significantly higher placement score and ball speed than both the LI-BFRT and HI-RT groups (adjusted p < 0.001), respectively (Table 12).

**TABLE 11:** Pre- and post-test results of forehand backspin loop effect in three groups.

Variable	Group	Pre-test	Post-test	*p*-value	Interaction effect			Cohen’s d	95% CI		Change ratio (%)
					F (Aram et al., 2024; 147)	p-value	partial η2		Lower	Upper	
placement (points)	CB-RT	2.28 ± 0.70	3.32 ± 1.02	0.000**	5.579	0.005**	0.071	1.19	−1.37	−0.71	45.61%
	LI-BFRT	2.20 ± 0.64	2.48 ± 0.65	0.095				0.43	−0.61	0.05	12.72%
	HI-RT	2.18 ± 1.01	2.66 ± 0.79	0.005**				0.53	−0.81	−0.15	22.01%
Ball speed (m/s)	CB-RT	12.13 ± 1.46	14.32 ± 1.52	0.000**	16.844	0.000**	0.186	1.47	−2.687	−1.711	18.06%
	LI-BFRT	12.22 ± 1.47	12.49 ± 1.24	0.265				0.20	−0.757	0.209	2.21%
	HI-RT	12.31 ± 1.59	13.04 ± 1.41	0.003**				0.49	−1.213	−0.247	5.93%

Pre-, before training; Post-, after training; *p < 0.05, **p < 0.01.

**TABLE 12:** Between-group post-hoc pairwise comparisons for forehand backspin loop effect among the three groups.

Variable	Comparison	Δ Mean difference	95% CI		Adjusted p-value	Cohen’s d
			Lower	Upper		
Placement (points)	CB-RT vs. LI-BFRT	0.76	0.435	1.245	0.000**	0.98
	CB-RT vs. HI-RT	0.56	0.255	1.065	0.000**	0.72
	LI-BFRT vs. HI-RT	−0.20	−0.585	0.225	0.849	−0.25
Ball speed (m/s)	CB-RT vs. LI-BFRT	1.92	1.152	2.508	0.000**	1.32
	CB-RT vs. HI-RT	1.46	0.605	1.961	0.000**	0.87
	LI-BFRT vs. HI-RT	−0.46	−1.225	0.131	0.158	−0.41

*p < 0.05, **p < 0.01.

## Discussion

This study conducted an 8-week strength training intervention protocol with three training sessions per week to comparatively analyze the training effects of the CB-RT, LI-BFRT, and HI-RT on knee isokinetic muscle strength, vertical jump performance, and stroke effect. The results indicated that the CB-RT group exhibited a greater improvement trend in all measured indicators. The LI-BFRT and HI-RT groups exhibited differential improvement characteristics across indices. The LI-BFRT group exhibited a greater improvement trend than the HI-RT group in SJ-related indicators and stroke effect of forehand topspin loop, while the HI-RT group exhibited a greater improvement trend than the LI-BFRT group in knee isokinetic muscle strength, CMJ-related indicators, and stroke effect of forehand backspin loop.

### Advantages of CB-RT

CB-RT serves as a multimodal training protocol, which can achieve significant improvements in lower limb muscle strength and stroke effect among table tennis athletes. Such optimal adaptive effects are difficult to obtain through single-mode LI-BFRT or HI-RT alone. Essentially, these outcomes can be attributed to the complementary advantages of the two training modalities, which facilitate the coordinated development of maximal strength and explosive force in the lower limbs. The present findings are consistent with those of previous studies, which have confirmed that CB-RT can effectively enhance athletes’ lower limb strength (Jiang et al., 2025; Sarfabadi et al., 2023). Sarfabadi et al. reported that this CB-RT protocol could effectively improve lower limb muscle strength and explosive force in long jump athletes (Sarfabadi et al., 2023). Jiang et al. also demonstrated that the CB-RT significantly enhanced lower limb muscle strength, vertical jump performance, and PRFD in wrestlers (Jiang et al., 2025).

From a physiological perspective, HI-RT induces neuromuscular adaptations by imposing greater mechanical tension (Su et al., 2025), thereby effectively improving the absolute muscle strength of knee flexion and extension. This provides a fundamental basis for the significant increase in peak torque of knee flexion and extension, with a change ratio ranging from 13.79% to 17.20% observed in the CB-RT group. In contrast, LI-BFRT limits limb blood circulation via external compression, inducing metabolic stress in skeletal muscles under low mechanical load. This may preferentially activate type II fast-twitch muscle fibers and improve the recruitment efficiency of motor units (Picabea-Arburu and Ozaeta-Beaskoetxea, 2025), ultimately enhancing the rapid contractile capacity and the PRFD of muscles. This may help explain why the CB-RT could activate muscular contraction more rapidly and sufficiently during the take-off phase of vertical jumps, achieving simultaneous improvements in jump height and PRFD. The change ratio of PRFD in the CB-RT group were 7.94% for SJ and 10.13% for CMJ, which were significantly higher than those in the other two groups. Jiang et al. confirmed that the training effects of CB-RT group were superior to those of isolated HI-RT group or LI-BFRT group (Jiang et al., 2025), which is highly consistent with the findings of the present study. Jiang further indicated that HI-RT compensates for the insufficient neuromuscular adaptation induced by low-load stimulation in LI-BFRT, whereas LI-BFRT makes up for the deficiency of HI-RT in stimulating the development of explosive force under low-load conditions, which further accounts for the results of this study. Nevertheless, Yasuda et al. found that a training regimen consisting of twice-weekly LI-BFRT plus once-weekly HI-RT could improve neuromuscular adaptation, however, its effects were not superior to isolated HI-RT (Yasuda et al., 2011). The inconsistency between their findings and the present results may be attributed to the difference in the intervention site: Yasuda et al. applied blood flow restriction to the upper limbs of athletes, while this study targeted the lower limbs. It is speculated that the adaptive responses to BFRT vary across different limb segments.

Existing studies mainly focused on the effects of BFRT on the improvement of general physical fitness indicators such as lower limb muscle strength and explosive force, while paying less attention to how such strength gains can be efficiently transferred to athletes’ sport-specific performance. The present study found that multimodal combined strength training enabled an efficient transfer from general physical fitness to table tennis-specific performance. The lower limbs play a vital role in the forehand loop of table tennis. As the starting point of the kinetic chain, lower limb force generation substantially affects racket velocity and ball speed (He et al., 2021). Although the present study did not directly measure force transfer along the kinetic chain, this theoretical framework may provide a tentative plausible explanation for the results observed in the CB-RT group. On the one hand, the enhancement of lower limb strength and explosive force enables athletes to achieve more stable lower limb support and sufficient propulsive force during the push-off phase when striking the ball, thereby improving ball speed (Liang et al., 2023). On the other hand, the improvement in neuromuscular control induced by HI-RT lays a foundation for precise force production. In addition, previous studies have demonstrated that LI-BFRT may improve athletes’ joint proprioception and enhance the perception and control of limb movement (Yeşilyaprak and Dere, 2025). Improved proprioception may potentially enable athletes to accurately perceive lower limb supporting posture during ball striking, adjust body alignment (Beckford et al., 2018), modulate force rhythm and movement amplitude in real time according to the incoming ball rotation, and correct movement deviations at the moment of impact, which may ultimately improve the accuracy of ball placement. This may reasonably explain the findings of the present study. The CB-RT group exhibited significant improvements in placement accuracy and ball speed for both forehand topspin and forehand backspin loop, with the highest growth rates among the three groups, reaching 45.61% and 18.06%, respectively.

The above results indicate that the multimodal training protocol adopted by the CB-RT group showed a more favorable improvement trend compared with single-mode LI-BFRT or HI-RT alone. Furthermore, this CB-RT possesses favorable training safety and sustainability, which is of important practical significance for elite table tennis athletes engaged in long-term high-intensity training. Under the premise of guaranteeing training effect, the combined strength intervention can reasonably distribute training load and reduce the risk of sports injuries and subsequent muscle fatigue induced by isolated HI-RT (Wang et al., 2023). Meanwhile, the complementation of metabolic stress and mechanical tension helps avoid the occurrence of training plateaus, thereby achieving continuous improvements in athletes’ physical fitness and sports-specific performance.

### The differences of effect between LI-BFRT and HI-RT

The results of this study revealed differential improvements in lower limb strength and stroke effect indicators between the LI-BFRT and HI-RT groups. The LI-BFRT group exhibited a greater improvement trend than the HI-RT group in SJ height, SJ PRFD during take-off, and stroke effect of forehand topspin loop, whereas the HI-RT group showed greater improvements in CMJ height, CMJ PRFD during take-off, and stroke effect of forehand backspin loop. These findings are consistent with the results reported by Jiang et al. (Jiang et al., 2025).

The core mechanical characteristic of SJ is rapid take-off without pre-squatting, which relies on the rapid recruitment and instantaneous force-generating capacity of the neuromuscular system. As a core technique for active attack and continuous rally in table tennis, the forehand topspin loop also requires athletes to complete trunk rotation and rapid ball striking within a short time (Iino and Kojima, 2009). The core physiological mechanism of LI-BFRT is to limit limb blood circulation via external compression. This could disrupt the size principle, in which motor units initially recruit type I slow-twitch muscle fibers followed by gradual recruitment of type II fast-twitch muscle fibers (Schoenfeld et al., 2023). Under ischemic conditions, more type II fast-twitch muscle fibers might be preferentially recruited (Manini et al., 2011), accompanied by the accumulation of muscle metabolic metabolites such as lactic acid (Kawada and Ishii, 2005). This process elevates the concentrations of growth hormone (GH) (Takano et al., 2005) and insulin-like growth factor-1 (IGF-1) (Aram et al., 2024), which may partially explain the observed gains in muscular explosive force. While the present data cannot directly verify these mechanisms, the observed improvements in SJ jump height (4.12%) and SJ PRFD (7.01%) in the LI-BFRT group align with the hypothesis that the aforementioned physiological responses may have contributed to these gains, which may in turn transfer to enhanced placement score (32.8%) and ball speed (6.34%) for the forehand topspin loop. Furthermore, LI-BFRT might help mitigate the muscular stiffness often induced by HI-RT, thereby potentially maintaining the limb flexibility and coordination required for effective ball striking.

CMJ performance relies on muscle elastic potential energy accumulated during the pre-squat phase, the capability of rapid stretch-shortening cycle from eccentric to concentric contraction, and maximal muscle strength. Its force production process requires prior energy accumulation followed by rapid release during the take-off phase. During the forehand backspin loop, athletes need to actively lower the center of gravity, store strength through lower limb eccentric contraction, and then generate force synchronously to counteract the rotation of incoming balls, which places higher demands on maximal strength and eccentric muscle capacity. The core advantage of HI-RT lies in the improvement of maximal muscle strength and the efficiency of the stretch-shortening cycle, as well as enhanced energy storage during the pre-squat movement. Accordingly, the HI-RT group achieved significant improvements in CMJ jump height (5.31%) and CMJ PRFD (6.96%), which were effectively transferred to increases in placement accuracy (22.01%) and ball speed (5.93%) during forehand backspin loop.

In summary, the discrepancies in the lower limb muscle strength and stroke effect between LI-BFRT and HI-RT essentially stem from differences in their physiological mechanisms and training emphases. These two training modes are differentially matched with the force generation characteristics of distinct table tennis specific techniques, ultimately resulting in varied improvements in lower limb muscular strength and stroke effect. The present findings provide a valuable reference for the formulation of targeted training protocols for table tennis athletes.

## Conclusion

1. The 8-week CB-RT can significantly improve the knee isokinetic muscle strength, vertical jump performance, and stroke effect of table tennis athletes. Furthermore, the effect of CB-RT exhibited a greater improvement trend than that of isolated LI-BFRT or HI-RT alone.
2. LI-BFRT exhibited superior improvements in SJ height, SJ PRFD during the take-off, and stroke effect of forehand topspin loop.
3. HI-RT yielded more pronounced enhancements in the knee isokinetic muscle strength, CMJ height, CMJ PRFD during the take-off, and stroke effect of forehand backspin loop.

## Practical implications

1. Incorporating CB-RT into the routine strength training of table tennis players may be of practical value. An intervention protocol comprising twice-weekly LI-BFRT sessions combined with one HI-RT session may be considered as a potentially effective strategy. This strategy may enhance lower limb maximal strength, explosive force, and stroke effect via the synergistic stimulation of mechanical tension and metabolic stress.
2. Based on the present exploratory findings, it may be suggested that targeted strength training protocols may be implemented according to the force generation characteristics of different table tennis techniques. To improve the stroke effect of forehand topspin loops, the proportion of LI-BFRT may be increased to facilitate muscle rapid contraction and boost instantaneous force production capacity; to potentially enhance the stroke effect of forehand backspin loops, the proportion of HI-RT may be raised to enhance maximal muscle strength and the efficiency of eccentric to concentric contraction.
3. LI-BFRT should be performed under professional supervision to avoid discomfort or sports injuries caused by inappropriate pressure settings or non-standard operation.

## Limitations

Several limitations should be acknowledged. First, the small sample size (n = 30, 10 per group) may reduce the statistical power and increase the risk of Type II errors. Second, although sex distribution was balanced across groups, the sample size (n = 10 per group) was insufficient for sex-stratified analyses. Therefore, this study is underpowered to investigate sex as a potential effect modifier. Third, diet and recovery were not monitored, although these factors may influence muscular adaptation. Fourth, alternative training frequency regimens (e.g., one LI-BFRT session combined with two HI-RT sessions per week) were not examined and warrant future investigation. Fifth, the generalizability of our findings to other populations requires further validation, as the present sample comprised only highly trained Chinese table tennis players. Finally, the absence of long-term follow-up precludes evaluation of sustained training effects. Future studies should address these issues with larger samples, sex-specific analyses, better covariate control, and delayed post-tests.

## Data Availability

The original contributions presented in the study are included in the article/supplementary material, further inquiries can be directed to the corresponding author.
